# Hydrodynamic Interactions and Entanglements of Polymer Solutions in Many-Body Dissipative Particle Dynamics

**DOI:** 10.3390/polym8120426

**Published:** 2016-12-09

**Authors:** Xin Yong

**Affiliations:** Department of Mechanical Engineering, State University of New York at Binghamton, Binghamton, NY 13902, USA; xyong@binghamton.edu; Tel.: +1-607-777-4981

**Keywords:** polymer solutions, many-body force law, Flory–Huggins theory, Zimm dynamics, entanglements

## Abstract

Using many-body dissipative particle dynamics (MDPD), polymer solutions with concentrations spanning dilute and semidilute regimes are modeled. The parameterization of MDPD interactions for systems with liquid–vapor coexistence is established by mapping to the mean-field Flory–Huggins theory. The characterization of static and dynamic properties of polymer chains is focused on the effects of hydrodynamic interactions and entanglements. The coil–globule transition of polymer chains in dilute solutions is probed by varying solvent quality and measuring the radius of gyration and end-to-end distance. Both static and dynamic scaling relations for polymer chains in poor, theta, and good solvents are in good agreement with the Zimm theory with hydrodynamic interactions considered. Semidilute solutions with polymer volume fractions up to 0.7 exhibit the screening of excluded volume interactions and subsequent shrinking of polymer coils. Furthermore, entanglements become dominant in the semidilute solutions, which inhibit diffusion and relaxation of chains. Quantitative analysis of topology violation confirms that entanglements are correctly captured in the MDPD simulations.

## 1. Introduction

Because of the fast evolution of polymer science and nanotechnologies, polymeric materials with precisely tailored functionalities, such as block copolymer thin films and polymer nanocomposites, become central to a wide range of emerging applications. In order to achieve versatile properties and functionalities of the macroscopic materials, polymeric building units with increasingly smaller sizes and different types of chemistry are integrated into more and more complex structural hierarchy in controlled and synergistic manners. This demand challenges manufacturing processes, in which one must close the gap between adjusting macroscopic characteristics and controlling micro-nanostructures of building blocks.

Among many existing methods for polymer synthesis and processing, solution deposition techniques (e.g., drop casting, dip coating, spin coating, electrospray, and printing) [1,2,3,4,5,6] are frequently employed due to the ease of implementation and low cost. Namely, an initial liquid precursor with solutes and/or dispersed moieties transforms into a solid material upon evaporation. The thermodynamics and kinetics of the precursor solutions during the drying process govern the final structure of the system [7,8,9,10,11]. However, the complex interplay among polymer, solvent, and other components in the evaporating mixtures is not fully understood; in-situ experimental characterization of non-equilibrium multi-component systems across multiple length and time scales is still difficult.

Computational simulation is appealing for elucidating the intricate polymer–polymer and polymer–solvent interactions and revealing detailed dynamics of the multi-component systems on the molecular scale. Dissipative particle dynamics (DPD) emerges as a popular mesoscopic technique for modeling polymer solutions/melts and probing their structural and rheological properties [12,13,14,15,16,17,18,19,20,21,22,23,24,25,26,27,28,29]. A recently developed variation, many-body dissipative particle dynamics (MDPD), further extends DPD’s capabilities to producing the liquid–vapor coexistence and simulating evaporation process [30,31,32,33,34,35], which is a promising step toward modeling solution-based processing of polymeric materials. Despite many successful MDPD simulations [35,36,37,38,39,40], there is no report on the parameterization of MDPD interaction parameters [37,41] for the liquid–vapor coexistent systems. More importantly, a comprehensive study of the conformation and dynamics of polymer chains in solutions is lacking.

This work seeks to establish a general mapping of MDPD parameters onto the Flory–Huggins *χ* parameter for the liquid state with coexisting vapor, which enables physical representations of multi-component, multi-phase polymeric systems, e.g., polymer solutions, copolymers, and polymer blends. Based on the obtained relation, polymer solutions with a full range of concentration and solvent quality are modeled using MDPD, followed by systematic characterization of the structural and dynamic properties of solvated polymer chains. The analysis is focused on revealing hydrodynamic interactions and quantifying entanglements of chains.

The present article is organized as follows: Section 2 briefly reviews the MDPD method and describes the details of the polymer simulations. Section 3.1 presents the parameterization of the MDPD interactions based on the mean-field Flory–Huggins theory. In Section 3.2, the conformation and dynamics of the polymer chains in dilute solutions with varying solvent quality are probed and the simulation results are compared to the Zimm model. Section 3.3 focuses on semidilute solutions and the effect of polymer volume fraction on the chain morphology and dynamics, through which the roles of hydrodynamic interactions and chain entanglements are elucidated. Finally, a summary of the important results and conclusions is presented in Section 4.

## 2. Methods

Many-body dissipative particle dynamics [30,32,33,34,41,42] is used to model polymer solutions with volume fractions ranging from 0.008 to 0.7, representing dilute solutions to semidilute solutions. MDPD is a mesoscopic particle-based method that can effectively model multi-phase, multi-component systems and captures correct hydrodynamic behavior [35,39,40,43,44,45,46,47,48,49]. In MDPD, a volume of fluid is modeled by coarse-grained beads, and each bead represents a cluster of molecules. The evolution of the entire system over time is dictated by the motion of beads, which is governed by Newton’s equation of motion, $mdv_{i}/dt=f_{i}$. The thermodynamics and transport properties of the system are determined statistically via the ensemble of MDPD beads.

The force acting on each bead *i* from neighboring beads consists of three parts $f_{i}\left(t\right)=\sum \left(F_{ij}^{C}+F_{ij}^{D}+F_{ij}^{R}\right)$, each of which is pairwise additive. The three terms, respectively, describe the conservative, drag, and random forces. The sum runs over all beads *j* within a cutoff radius $r_{c}$ from bead *i*. The main advantage of MDPD compared to standard DPD is its ability to produce the coexistence of liquid and vapor phases in coarse-grained fluids, which is achieved by introducing a long-range attractive force that is responsible for surface tension. In particular, the conservative force $F_{ij}^{C}$ is given by $F_{ij}^{C}=A_{ij}\left(1-r_{ij}/r_{c}\right){\hat{r}}_{ij}+B_{ij}\left({\bar{\rho }}_{i}+{\bar{\rho }}_{j}\right)\left(1-r_{ij}/r_{d}\right){\hat{r}}_{ij}$ with $r_{ij}=\left|r_{i}-r_{j}\right|/r_{c}$ and ${\hat{r}}_{ij}=\left(r_{i}-r_{j}\right)/\left|r_{i}-r_{j}\right|$ [32]. Here, The attraction strength $A_{ij}<0$, the repulsion strength $B_{ij}>0$, and the repulsion range $r_{d}<r_{c}$, making repulsion short-range and attraction long-range. The repulsive term depends on local densities ${\bar{\rho }}_{i}$ and ${\bar{\rho }}_{j}$ as well as inter-bead distance $r_{ij}$; the attractive term depends only on the distance. The interaction parameters $A_{ij}$ and $B_{ij}$ are given in terms of $k_{B}T/r_{c}$, where $k_{B}T$ is the Boltzmann constant and *T* is the temperature of the system. The local density for each particle is defined as ${\bar{\rho }}_{i}=\sum_{j\neq i}15/2\pi r_{d}^{3}{\left(1-r_{ij}/r_{d}\right)}^{2}$ [32]. This many-body conservative force was shown to produce cubic pressure-density equations of state with van der Waals loop, thereby permitting the liquid–vapor coexistence with a sharp interface [30,32,34,35]. Notably, the soft-core repulsive term allows a degree of overlap between beads, in contrast to hard-core potentials (e.g., the Lennard–Jones potential) in which the repulsion diverges at zero inter-bead distance. Thus, larger time steps than those typically used in molecular dynamics (MD) simulations, which commonly involve hard-core potentials, can be applied in the MDPD simulations. Together with the coarse-grained representation of multi-component, multi-phase systems, MDPD can capture physical phenomena occurring on relatively larger length and time scales than those normally captured by MD. These features make MDPD an ideal computational tool to resolve the polymer dynamics in liquid–vapor multi-phase systems.

The drag force is $F_{ij}^{D}=-\lambda \omega_{D}\left(r_{ij}\right)\left({\hat{r}}_{ij}\cdot v_{ij}\right){\hat{r}}_{ij}$, where λ is a simulation parameter related to the viscosity arising from the interactions between the constituent beads of fluid. $\omega_{D}$ is a weight function satisfying $\omega_{D}=0$ at $r_{ij}=r_{c}$, and the relative velocity is $v_{ij}=v_{i}-v_{j}$. The random force is $F_{ij}^{R}=\sigma \omega_{R}\left(r_{ij}\right)\xi_{ij}{\hat{r}}_{ij}$, where $\xi_{ij}$ is a zero-mean Gaussian random variable of unit variance and σ is the amplitude of the noise. The fluctuation-dissipation theorem relates σ to λ as $\sigma^{2}=2k_{B}T\lambda$ [50]. Finally, the weight functions take the following form: $\omega_{D}\left(r_{ij}\right)=\omega_{R}{\left(r_{ij}\right)}^{2}={\left(1-r_{ij}/r_{c}\right)}^{2}$ for $r_{ij}<r_{c}$ [15]. The combination of drag force and random force serves as a thermostat applied on the MDPD system for producing the canonical (NVT) ensemble [14,15,50]. All three forces act on pairs of neighboring beads such that momentum is conserved locally, and hydrodynamic behavior emerges in relatively small systems [12,14]. 

The equation of motion is integrated in time using the velocity-Verlet algorithm. The simulation takes $r_{c}$ as the characteristic length scale with a dimensionless value as $r_{c}=1$. The corresponding energy scale $k_{B}T$ at room temperature is chosen as the characteristic energy. Thus, $k_{B}T=1$ at T=25 °C is considered in this work. The characteristic mass is defined as the mass of a MDPD bead and takes the dimensionless value of 1. The characteristic time scale is then defined as $\tau =\sqrt{mr_{c}^{2}/k_{B}T}=1$. A time step Δt=0.01 is used for all simulations characterizing static and dynamic properties of polymer [15,51]. Unless otherwise stated, the value of λ is chosen as 4.5 to obtain a relatively rapid equilibration of the system temperature and to ensure the numerical stability of the simulations for the specified time step. All simulations are performed by modifying and extending the particle dynamics software code LAMMPS [52]. 

In this work, the MDPD non-bonded parameters of $A_{ij}=-40$ and $B_{ij}=40$ are used for any two beads of the same type (i.e., solvent–solvent or polymer–polymer interactions). Notably, MDPD applied to multi-component systems requires a constant $B_{ij}$ to ensure a conservative many-body force law [53]. Thus, the attraction parameter $A_{ij}$ is adjusted to control the polymer–solvent interactions, which can be mapped to the Flory–Huggins *χ* parameter as detailed below. The repulsion cutoff radius is set as $r_{d}=0.8$ for all non-bonded interactions. As used in our previous study, this parameter set yields a liquid–vapor system with a liquid number density of 3.926 and viscosity of 3.510 [35]. Therefore, the total density of polymer solutions is set to $\rho_{\mathrm{sys}}=3.926$ for all simulations conducted in this study.

The widely used bead-spring model is applied to represent flexible polymer chains. Groups of repeating units in a linear polymer are modeled as consecutive MDPD beads connected by Hookean springs, whose potential is given by $E_{\mathrm{bond}}=\frac{1}{2}K_{b}{\left(r_{ij}-r_{0}\right)}^{2}$. Here, $K_{b}=128$ is the elastic constant, $r_{ij}$ is the distance between bonded beads *i* and *j*, and $r_{0}$ is the equilibrium bond distance. An equilibrium bond distance of 0.685 is chosen such that the mean distance between connected beads coincides with the position of the first neighbor peak in the radial distribution function of the MDPD liquid with density 3.926. Initial configuration of each polymer chain is created by a self-avoiding random walk on a face-centered-cubic (fcc) lattice superimposed on the simulation box [54,55]. Polymer beads in a chain are consecutively inserted onto the lattice in a stepwise manner, mimicking chain-growth polymerization. The probability of successful insertion and chain growth depends on the generated bond angle and the density of unoccupied sites. Once the polymers are constructed, the corresponding number of solvent beads are added to the system at randomly positions to solvate the polymer and obtain the desired polymer volume fraction. 

The dimensions of the simulation box vary from 10 × 10 × 10 to 40 × 40 × 40, with periodic boundary conditions imposed in all three directions. The total number of beads in our simulations ranges from 3000 to 251,264. The number of beads per chain is varied from *N* = 5 to 140. In order to minimize the finite-size effect on the equilibrium properties of the system [21,22,23], the length *L* of the cubic simulation box satisfies the condition $L/R_{g}>5$, where $R_{g}$ is the radius of gyration of the polymer chains. Each simulation system is typically equilibrated for 5 × 10^5^ time steps prior to a production run of at least 5 × 10^5^ time steps.

## 3. Results and Discussion

### 3.1. Parameterization of MDPD Parameters

**Figure 1:**
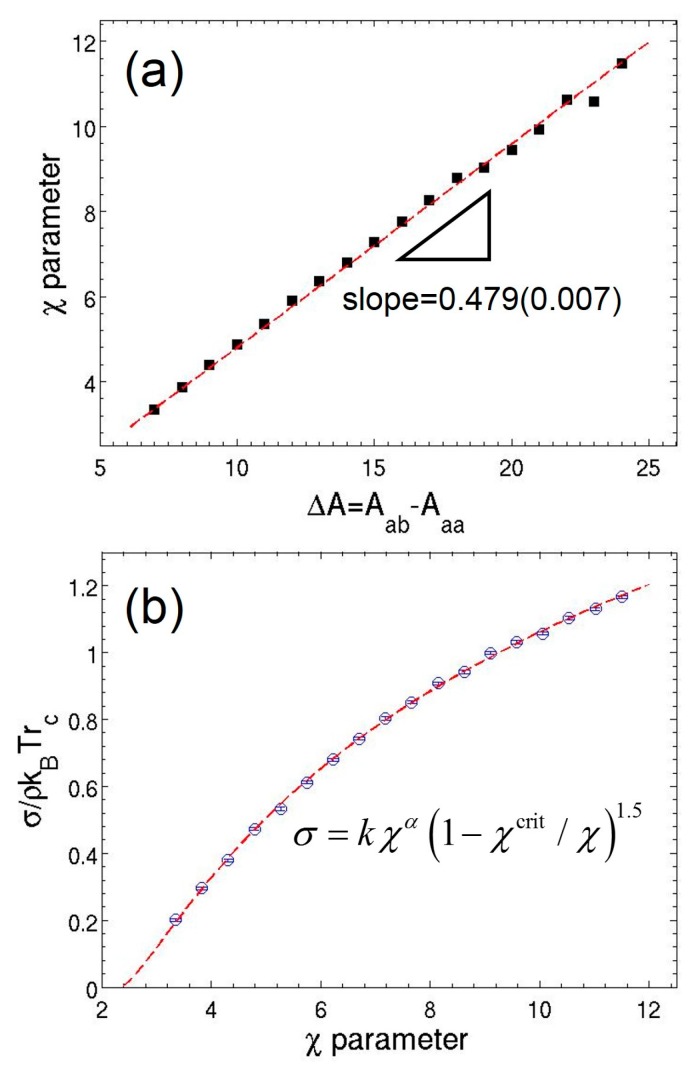
(**a**) Relation between effective *χ* parameter and excess repulsion between unlike species in a binary mixture of monomers defined as $\Delta A=A_{\mathrm{ab}}-A_{aa}$, where *a* and *b* represent the types of interacting beads. The dash line is the least-square linear fitting. (**b**) Simulated surface tension of the binary mixture as a function of *χ* parameter. The smooth curve is a fit to a 3/2 power law with a non-classical critical point.

In order to represent physical systems of specific chemistry, one must establish a rigorous approach to the parameterization of the interaction parameters. Unlike the commonly applied DPD method [15,56], to date only a few MDPD studies have reported general methods for obtaining a corresponding parameter set capable of reproducing the thermodynamic properties of experimental fluids [37,41]. The central idea of the MDPD parameter derivation is mapping the configurational part of the free energy density of MDPD binary mixtures to the mixing free energy density from the mean-field Flory–Huggins lattice theory [57], similar to the approach applied to DPD method [15]. Due to the no-go theorem of MDPD ($B_{aa}=B_{bb}=B_{ab}$ for a binary mixture of *a* and *b* fluids), the cubic repulsive term in the MDPD equation of state of a single component fluid [32] $p=\rho k_{B}T+\alpha A\rho^{2}+2\alpha Br_{d}^{4}\left(\rho^{3}-c\rho^{2}+d\right)$ does not contribute to the configurational part, where *α*, *c*, and *d* are fitting parameters [32]. Thus, the mapping yields the same expression of the Flory–Huggins *χ* parameter as the one for DPD, $\chi =2\alpha \left(\rho_{a}+\rho_{b}\right)\left(A_{\mathrm{ab}}-A_{aa}\right)/k_{B}T=2\alpha \rho_{\mathrm{sys}}\Delta A$. A set of simulations of binary mixtures of monomers is carried out to establish the *χ*-Δ*A* relation, where only the cross attraction parameter $A_{\mathrm{ab}}$ is adjusted from −14 to −7. The *χ* parameter is calculated from the density profiles of the phase-separated systems. Only systems showing strong phase separation are sampled because the Flory–Huggins mean-field expression breaks down for systems with small *χ* [15]. A good linear relation between *χ* and excess repulsion Δ*A* is confirmed in Figure 1a; the fitting results in χ=(0.479±0.007)ΔA for $\rho_{\mathrm{sys}}=3.926$. Notably, the MDPD method can be readily extended to simulate ternary systems given that the parameterization of the self and cross interaction parameters is carried out in a rigorous way. In addition to the Flory–Huggins approach employed in this work, Hildebrand solution theory approach [58,59] and more advanced ab-initio methodologies [60] may be adapted in MDPD to simulate ternary mixtures of beads dissimilar in both size and chemistry.

The surface tension of the binary mixtures can be also determined in the simulations. The Irving–Kirkwood expression is used to measure surface tension, given as $\sigma =\frac{L_{z}}{2}\langle p_{zz}-\frac{1}{2}\left(p_{xx}+p_{yy}\right)\rangle$, where $p_{xx}$, $p_{yy}$, and $p_{zz}$ are the diagonal components of the microscopic pressure tensor [61] and $L_{z}$ is the dimension of the simulation box in the *z* direction. Here, the *z* direction is normal to the time-averaged interfaces. The factor of 1/2 arises from the existence of two interfaces in the periodic simulation domain. The angular bracket represents ensemble average. The dependence of surface tension on the *χ* parameter is subsequently compared to the classical van der Waals theory $\sigma ∼\chi^{\alpha }{\left(1-\chi^{\mathrm{crit}}/\chi \right)}^{1.5}$. Figure 1b shows good agreement between the simulation results and the theory. The variation of surface tension is given by the fitting $\sigma ∼\left(0.82\pm 0.04\right)\chi^{0.29\pm 0.02}{\left[1-\left(2.34\pm 0.03\right)/\chi \right]}^{1.5}$. Consistent with the DPD results [15], the extrapolation of the surface tension of the MDPD fluids leads to a non-classical critical point of a value of 2.34 higher than the theoretical value $\chi^{\mathrm{crit}}=2$. Nevertheless, the critical point found from the MDPD simulations agrees well with the one obtained in the DPD simulations.

### 3.2. Dilute Polymer Solutions for Different Solvent Quality

**Figure 2:**
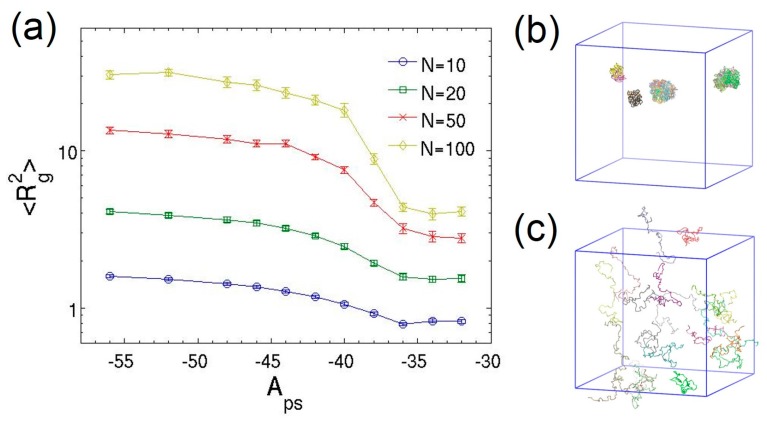
(**a**) Mean-square radius of gyration as a function of solvent quality quantified by $A_{\mathrm{ps}}$ for dilute polymer solutions with different chain lengths. Snapshots of dilute polymer solutions with chain length *N* = 100 in the: (**b**) globule; and (**c**) expanded coil states. Colors represent different chains. The lines are only guides to the eyes. The error bars represent the standard deviations of the ensemble averages.

Dilute polymer solutions with a volume fraction ϕ≈0.008 are investigated. Each system has *M* chains with *N* beads per chain. The inter-molecular interactions between different chains are present in the system, closely representing physical systems. The number of chains is adjusted accordingly to keep the polymer volume fraction constant when the chain length is varied. The solvent quality is controlled by tuning the polymer–solvent attraction parameter $A_{\mathrm{ps}}$, which determines the polymer–solvent Flory–Huggins parameter $\chi_{\mathrm{ps}}$ according to the linear relation obtained above. The mean-square radius of gyration of the polymer chains and their end-to-end distance are calculated to characterize the equilibrium structure of polymer chains, defined by $\langle R_{g}^{2}\rangle =\langle \left(1/N\right)\sum_{i=1}^{N}{\left(r_{i}-r_{\mathrm{com}}\right)}^{2}\rangle$ and $\langle R_{1N}^{2}\rangle =\langle {\left(r_{1}-r_{N}\right)}^{2}\rangle$, respectively. Here, $r_{i}$ is the position of the *i*th bead in a polymer chain and $r_{\mathrm{com}}$ is the center-of-mass position of the chain. The coil–globule transition of the flexible chains having different lengths is monitored as the solvent quality varies. Figure 2 clearly shows the characteristic collapse of polymer chains from an expanded coil state to a globule state as $A_{\mathrm{ps}}$ decreases, modeling the solvent quality change from good to poor. The observed continuous coil–globule transitions are consistent with theory predictions [62,63], where longer chains exhibit much more pronounced change in size. The spatial distribution of chains also reveals that the conformation transition is accompanied by the phase separation due to inter-molecular interactions. In particular, multiple collapsed chains aggregate into a bigger globule as shown in Figure 2b. Figure 2a also indicates that the Flory theta point of the transition corresponding to the ideal chain behavior does not coincide with the athermal condition of $A_{\mathrm{ps}}=A_{\mathrm{ss}}=A_{\mathrm{pp}}=-40$.

**Figure 3:**
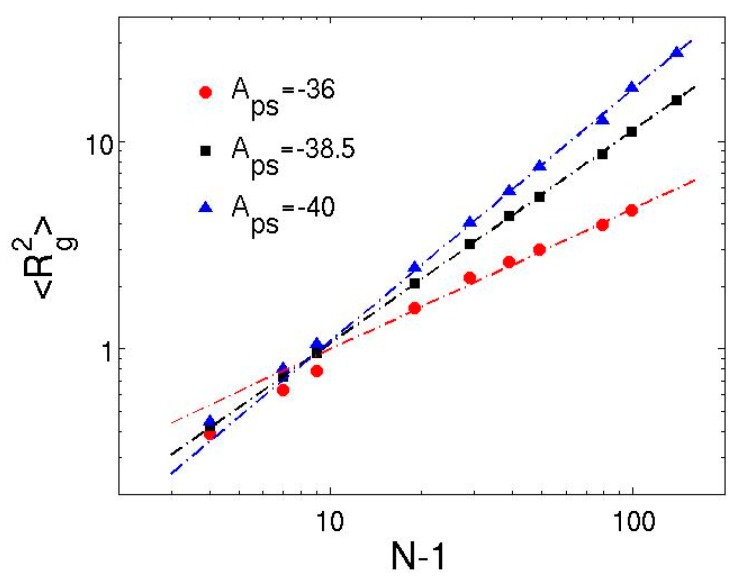
Log–log plot of mean-square radius of gyration as a function of the number of Hookean springs in each chain for different solvent quality. The dash-dot lines correspond to the power-law fittings of simulation data. The error bars are smaller than the symbols.

To explicitly analyze the correspondence of solvent quality to $A_{\mathrm{ps}}$, Figure 3 plots the variation of mean-square radius of gyration as the chain length increases on the log–log scale for three different values of $A_{\mathrm{ps}}$. The linear behavior of $\langle R_{g}^{2}\rangle$ as a function of bond number confirms that the MDPD results can be well described by the classical scaling law [64,65], $\langle R_{g}^{2}\rangle ∼{\left(N-1\right)}^{2\nu }$, where *ν* is the Flory scaling exponent. The good agreement between the simulation data and the theory indicates that, at low polymer volume fractions, the presence of polymer–polymer inter-molecular interactions have negligible effect on the polymer conformation. The power-law fitting of the simulation data for the athermal solvent ($A_{\mathrm{ps}}=-40$) yields $\langle R_{g}^{2}\rangle =\left(0.066\pm 0.008\right){\left(N-1\right)}^{1.22\pm 0.03}$. The end-to-end distance also obeys the scaling relation, and the fitting results in $\langle R_{1N}^{2}\rangle =\left(0.49\pm 0.09\right){\left(N-1\right)}^{1.17\pm 0.04}$. The scaling exponents obtained from both measurements are consistent: v=0.61±0.02 from the radius of gyration and v=0.59±0.02 from the end-to-end distance. The exponents imply that the chains exhibit good solvent behavior in the athermal solvent, where effective excluded volume interactions are imparted through the MDPD conservative force. This result is consistent with the DPD simulations of dilute solutions of flexible “bead-spring” chains [21,23], but is in contrast to recent characterization by Jamali et al. [41] for MDPD fluids, in which the athermal solvent leads to the ideal-chain exponent of 0.5.

**Figure 4:**
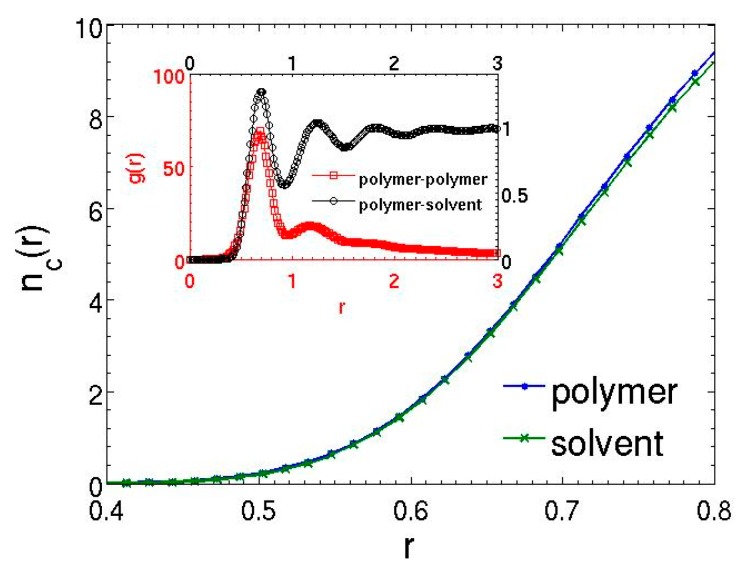
Time-averaged coordination numbers for polymer and solvent beads in the athermal solution for $A_{\mathrm{ps}}=-40$. The inset plots the time-averaged polymer–polymer and polymer–solvent radial distribution functions. The lines are only guide to the eyes.

A prominent feature of MDPD is the many-body nature of the repulsive part of the conservative force, which depends on the local density. Due to the athermal condition of the attractive force, the excluded volume effect must be induced by the difference in repulsions, leading to energetically favorable polymer–solvent interactions. Moreover, constant repulsion strength suggests that the preferential interaction between polymer and solvent beads is caused by the local density difference. In order to provide quantitative evidence, the structural property of the polymer solution in the athermal condition is further examined by the coordination numbers and radial distribution functions (the calculations include the bonded, nearest neighbor beads). Figure 4 shows the time-averaged coordination numbers of polymer and solvent beads up to the repulsion cutoff $r_{d}=0.8$. Although the polymer chains are formed by matching the equilibrium bond distance to the nearest neighbor distance of the solvent, the coordination number of the polymer beads is still slightly higher than that of the solvent beads, which is caused by the existence of the bonded neighbors for polymer beads. The local densities of a bead should scale with its coordination number. Thus, the repulsion between two polymer beads will be larger than between a polymer bead and a solvent bead, leading to unfavorable polymer–polymer interactions and the effective excluded volume effect. The radial distribution functions in Figure 4 also confirm that the polymer beads interact preferentially with the solvent beads.

The reduction of polymer–solvent attraction strength increases the *χ* parameter and cancels the excluded volume effect, leading to the collapse of the chains. The transition from good to poor solvent occurs near $A_{\mathrm{ps}}=-38.5$, where the scaling exponent for radius of gyration becomes v=0.51±0.01. The polymer coils behave like ideal chains in this theta solvent except for small *N*, as the ratio of the mean-square end-to-end distance to the mean-square radius of gyration $\langle R_{1N}^{2}\rangle /\langle R_{g}^{2}\rangle \approx 6$ (see Table 1). For even larger value of $A_{\mathrm{ps}}$, the scaling exponent decreases to v=0.29±0.03, corresponding to the poor solvent condition and the associated globule state. Notably, the behavior of short polymers in theta and poor solvents deviates from the power-law predictions. In Figure 3, the slope of the $\langle R_{g}^{2}\rangle$ curve is significantly higher than 0.3 for short chains in the poor solvent ($A_{\mathrm{ps}}=-36$). This implies that short chains are more extended than the ideal chains described in the theory, which is attributed to the intrinsic chain rigidity induced by the many-body repulsion. The results show that the scaling regime in MDPD generally requires chains having more than 10 beads. Thus, the effective persistence length of the bead-spring chains in MDPD is longer than that in DPD, where even chains with as few as five beads were found to follow the scaling relation well [20,22]. 

The excluded volume effect and associated entropic cost for solvation of polymer can be interpreted in the light of the scaled particle theory [66,67]. The theory considers a chain with *N* beads of diameter *d* in a solvent of beads with the same diameter, where the volume of a single bead is $v=\pi d^{3}/6$. The polymer chains do not interact in dilute solutions—each independently occupies a volume characterized by $R_{g}$. Therefore, the mean volume fraction of polymer is $\phi_{p}\propto Nd^{3}/R_{g}^{3}$ and the solvent volume fraction is $\phi_{s}=1-\phi_{p}$. Solvation of polymer requires work to form a cavity in the solvent to accommodate the solute molecules. Considering consecutive insertions of chain beads, the total work of cavity formation for a chain is given by $W_{p}\left(N,d\right)=\sum_{j=0}^{N-1}W_{j}\left(d\right)$, where *j* is the number of previously inserted beads. Upon the insertion of the *j*th bead, the chain occupies volume fraction $\phi_{p,j}=\phi_{p}j/N\propto dj/{\left(N-1\right)}^{3\nu }$, and the total occupied volume faction is $\phi_{j}=\phi_{p,j}+\phi_{s}$. The work for individual bead $W_{j}\left(d\right)$ is given by the scaled particle theory [67] as $W_{j}\left(d\right)/k_{B}T=\ln\left(1+y_{j}\right)+9y_{j}+15y_{j}^{2}/2+3y_{j}^{3}$, where the ratio of occupied to unoccupied volume is $y_{j}\equiv \phi_{j}\left(1-\phi_{j}\right)$. According this expression, the entropic cost for cavity formation increases as the solvent quality varies from good to poor, quantified by the scaling exponent *ν*. The increase of chain length *N* leads to smaller entropic cost regardless of the solvent quality.

**Figure 5:**
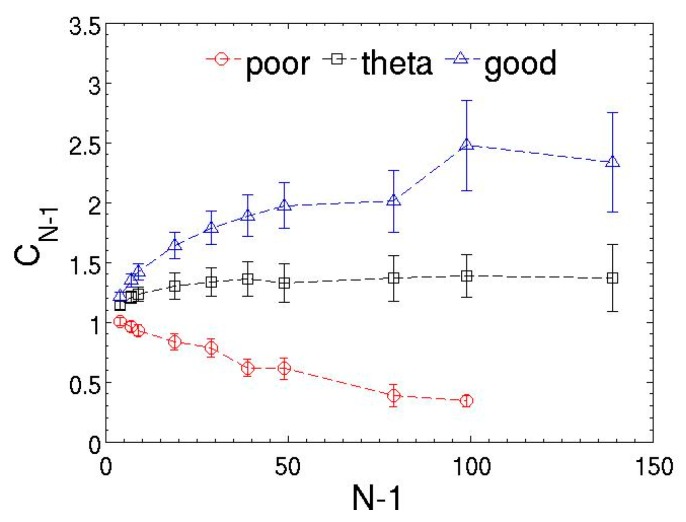
Characteristic ratio of the modeled chains as a function of chain length for different solvent quality. Poor, theta, and good solvents refer to the systems with polymer–solvent attraction parameter $A_{\mathrm{ps}}$ = −36, −38.5, and −40, respectively. The lines are only guides to the eyes. The error bars represent the standard deviations of the ensemble averages.

Another measurement of the conformation and stiffness of polymer chains is the dimensionless characteristic ratio, defined as the ratio of the measured mean-square end-to-end distance to the value of an ideal chain $C_{N-1}=\langle R_{1N}^{2}\rangle /\left[\left(N-1\right)\langle l^{2}\rangle \right]$. Here, $\langle l^{2}\rangle$ is the mean-square bond length. As the chain length increases, the characteristic ratio approaches a limiting value $C_{\infty }$ as shown in Figure 5. Namely, the characteristic ratio decreases in a poor solvent and increases in theta and good solvents. Table 1 tabulates the characteristic ratio of chains with different lengths in a theta solvent. The asymptotic value $C_{\infty }$ is approximately 1.4, which is slightly larger than the value of 1.27 in the DPD simulations [51]. This provides additional evidence of the enhanced excluded volume interactions caused by the MDPD force law compared to the DPD model, which effectively increases chain rigidity. 

**Figure 6:**
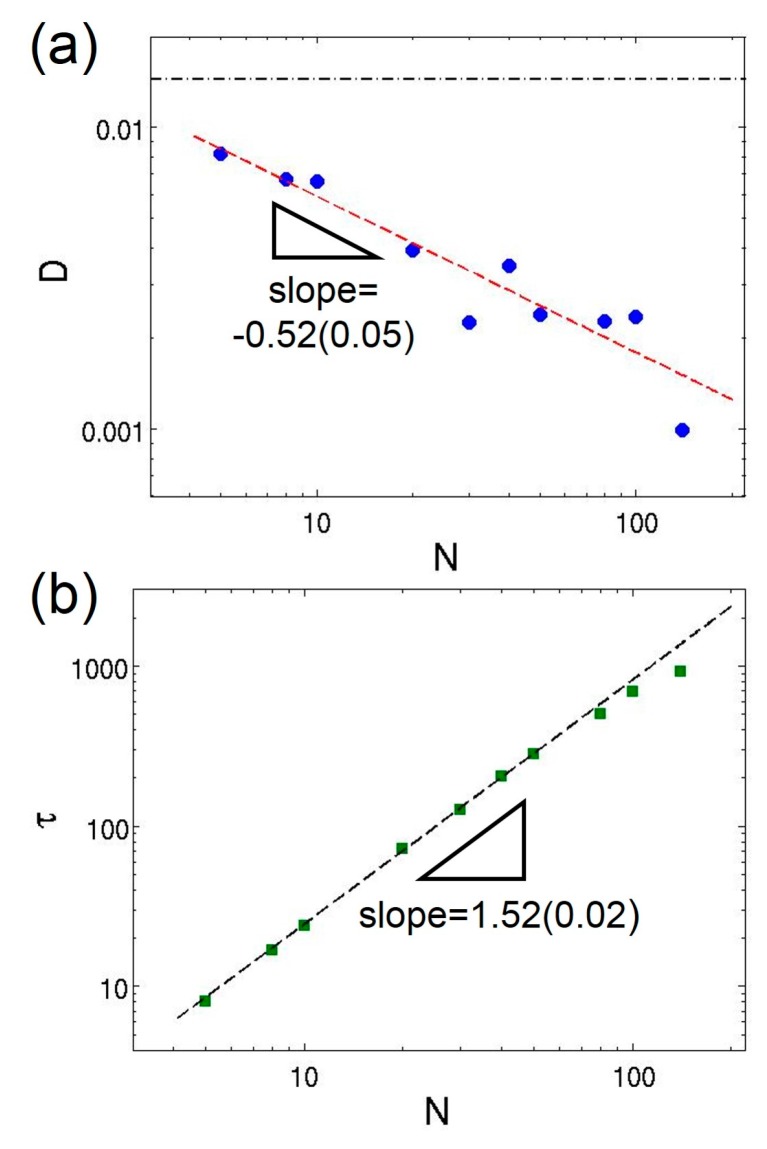
Log–log plots of: (**a**) the diffusion coefficient; and (**b**) the longest relaxation time as functions of chain length in the theta solvent. The black dash-dot line in (**a**) indicates the diffusion coefficient of the solvent beads. The dashed lines in (**a**,**b**) represent the power-law fittings of the data. The error bars are smaller than the symbols.

One major advantage of DPD and MDPD simulations over other coarse-grained techniques such as Monte-Carlo and self-consistent field theory is the ability to simulate dynamical systems. The dynamical properties of polymer chains in the theta solution are probed through diffusion coefficient and relaxation time. The diffusion coefficient can be measured by calculating the mean-square displacement of the center-of-mass coordinate for each chain, $\langle {\left[r_{\mathrm{com}}\left(t\right)-r_{\mathrm{com}}\left(0\right)\right]}^{2}\rangle$, and utilizing the Einstein relation, $\langle {\left[r_{\mathrm{com}}\left(t\right)-r_{\mathrm{com}}\left(0\right)\right]}^{2}\rangle =6Dt$. The value is extracted from the linear fitting of the mean-square displacement. Notably, the diffusion behavior of polymer can still suffer from strong finite-size effect even when $L/R_{g}>5$ [23], but the influence on the scaling law of the diffusion coefficient is negligible. Figure 6a shows the diffusion of polymer chains in MDPD simulations with a fitting $D∼N^{-0.52\pm 0.05}$ for the theta solvent. The scaling exponent is consistent with the Flory exponent v=0.51±0.01 obtained from the mean-square radius of gyration. The diffusion of polymer chains in dilute solutions obeys the Zimm model, $D∼N^{-\upsilon }$ [64]. Therefore, the MDPD simulations correctly capture hydrodynamic interactions considered in the Zimm model. In addition, the relaxation time is another important probe of the polymer dynamics, which can be extracted from the conformational autocorrelation functions. Herein, the autocorrelation function of the end-to-end vector of the chains $R_{1N}=r_{1}-r_{N}$ is calculated, defined as $C\left(t\right)=\langle R_{1N}\left(t+t_{0}\right)\cdot R_{1N}\left(t_{0}\right)\rangle /\langle R_{1N}^{2}\rangle$. The angle bracket means average over all chains and many time origins, $t_{0}$. The longest relaxation time τ is obtained by fitting the autocorrelation function to an exponential form, $C\left(t\right)=C_{0}\exp\left(-t/\tau \right)$. The relaxation times of polymer chains having different lengths in the theta solvent are shown in Figure 6b. Apparently, the linearity of the data point breaks down for long chain lengths, possibly due to the finite-size effect and interactions between image chains across the periodic boundaries. The best fitting of the relaxation time excluding chains of length N≥80 yields $\tau =\left(0.73\pm 0.06\right)N^{1.52\pm 0.02}$. The Zimm model predicts that $\tau ∼N^{3\nu }\sim N^{1.5}$ for theta solvents, while the Rouse model overestimates the relaxation time as $\tau ∼N^{2}$. Again, the simulation results confirm the Zimm dynamics for the modeled polymer chains.

### 3.3. Semidilute Solutions and Entanglements

In this section, the results of semidilute polymer solutions with volume fractions ranging from 0.02 to 0.7 are presented. The number of chains in this series of simulations is 100 and the chain length is 30. The chains of this length exhibit reptation behavior in previous simulations [51,68]. Due to considerably stronger density-dependent repulsion between MDPD beads than the standard DPD repulsion, an MDPD system exhibits thermodynamic behavior quite different from that of a standard DPD simulation [51]. Notably, the thermodynamics of polymer solutions with high volume fractions or even polymer melts is significant influenced by the MDPD force law. Consequently, the MDPD simulations typically require a stronger thermostat coupling to achieve stable temperature control. This demands elevated thermostat parameters *σ* and *λ* (obeying the fluctuation-dissipation relation). Using the standard parameter λ=4.5 (σ=3), the maximum temperature increase of 33% is observed in a polymer melt system with ϕ=1.0, which completely shifts the thermodynamic state of the system. For semidilute polymer solutions, a value of λ=50 (σ=10) is applied to maintain the temperature within 4% of the set value [51]. 

**Figure 7:**
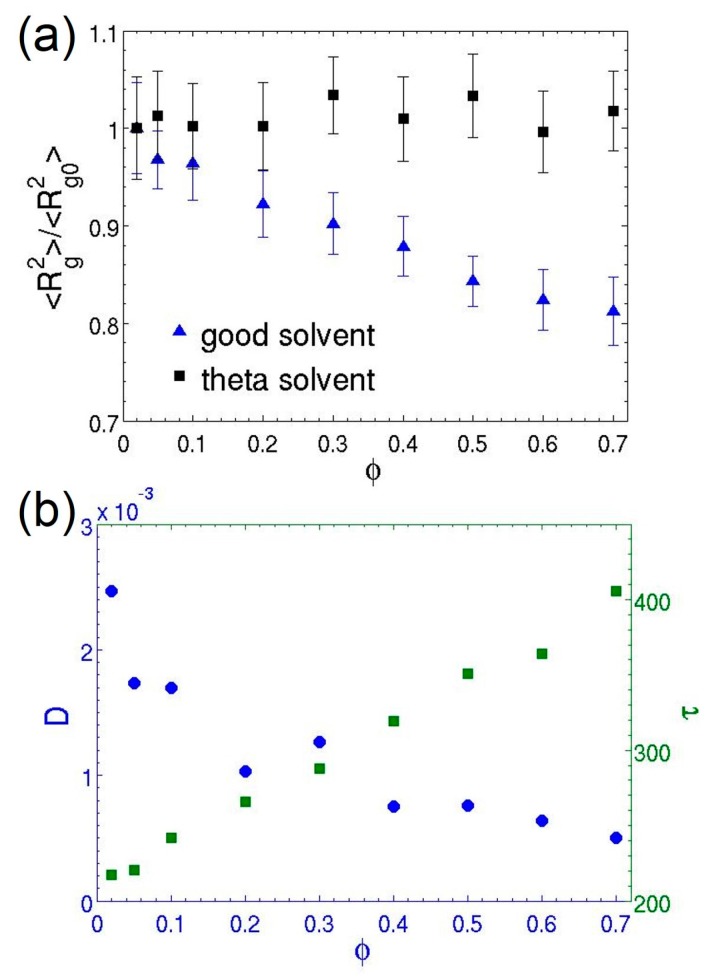
(**a**) Dependence of the normalized mean-square radius gyration (with respect to the values in the dilute limit $\langle R_{g0}^{2}\rangle$) of the N=30 polymer chains on their volume fraction for good ($A_{\mathrm{ps}}=-40$) and theta ($A_{\mathrm{ps}}=-38.5$) solvent conditions; (**b**) The diffusion coefficient and relaxation time of the chains in the theta solvent as functions of the volume fraction. The error bars represent the standard deviations of the ensemble averages, and their sizes are smaller than the symbols in (**b**).

Figure 7 shows the dependence of the polymer conformational and dynamic properties on the volume fraction in semidilute solutions. The excluded volume interactions are screened when the polymer chains overlap with each other at high volume fractions. Therefore, the swelling of the coils in a good solvent gradually diminishes as φ increases and can finally vanish in the melt. The reduction of the degree of swelling, which is characterized by the mean-square radius of gyration, is clearly depicted in Figure 7a. The simulation observation agrees well with the theoretical prediction. In contrast, the chain morphology in a theta solvent does not exhibit φ dependence, since the coils always behave as ideal chains in the theta condition.

**Figure 8:**
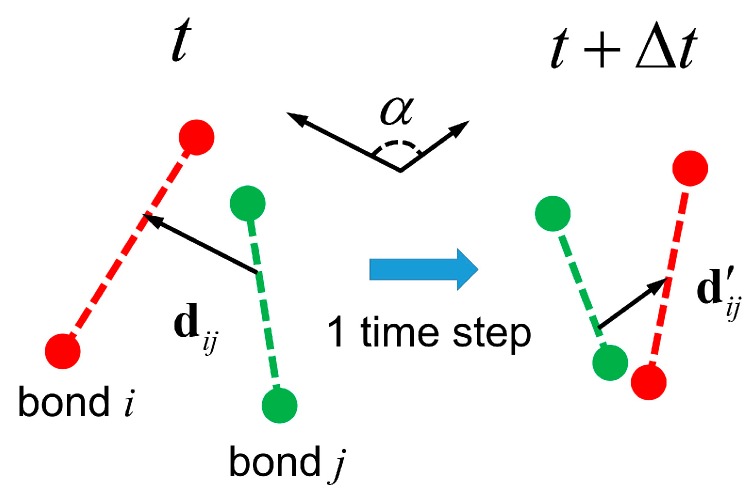
Schematic of a topology violation, where the minimum distance vector between bonds changes orientation by an angle larger than 90° in one time step. Red and green colors represent different bonds.

As the concentration increases, the polymer dynamics undergo a transition from the Zimm regime to the reptation regime, in which the onset of topological constraints (entanglements) between chains severely hinders thermal motion of polymers. Consequently, the diffusion and relaxation of chains are slowed down significantly, as shown in Figure 7b. The MDPD results show the relaxation time *τ* increases by a factor of 2 at the highest volume fraction investigated here. This increase is more pronounced than the factor of 1.4 observed in comparable DPD simulations [23]. However, the value is consistent with previous MD simulations [69]. Future analysis on entanglements is performed to provide additional insights into the dynamics behavior of polymer chains in the MDPD model. For coarse-grained simulations, there have been serious concerns about unphysical bond crossings of the polymer chains (referred to as topology violations below) associated with the use of soft-repulsive potentials [51,70,71,72,73,74,75]. An exemplary topology violation is illustrated in Figure 8. In order to accurately predict behavior of the polymers in semidilute and concentrated solutions, chain entanglements must be properly captured in the MDPD model.

**Figure 9:**
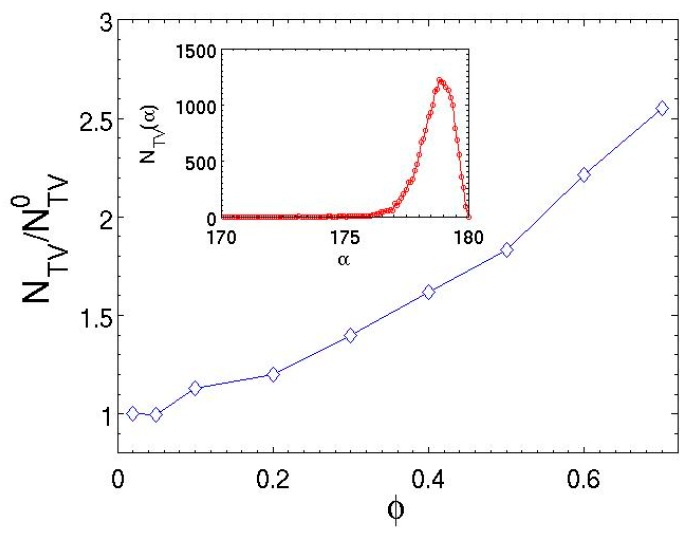
Relative number of topology violations as a function of polymer volume fraction in the good solvent with $A_{\mathrm{ps}}=-40$. The reference number of topology violation is for the dilute limit. The inset shows the distribution of the α angle between 170° and 180° for the topology violation events in the concentrated solution (ϕ=0.7) during a total simulation time of t=5000τ.

Quantitative measurement of topology violations is conducted by calculating the minimum distance vector between bonds, $d_{ij}$ [23,51,70,76]. The method has been detailed elsewhere [70]. The angle *α* between the two minimum distance vectors defined at time *t* and *t* + Δ*t* is given by the dot product, $\cos\alpha ={\hat{d}}_{ij}\left(t\right)\cdot {\hat{d}}_{ij}\left(t+\Delta t\right)$, where ${\hat{d}}_{ij}=d_{ij}/\left|d_{ij}\right|$ is the normalized unit vector. A topology violation is detected when the direction of ${\hat{d}}_{ij}=d_{ij}/\left|d_{ij}\right|$ changes by 90° or more over a single time step, i.e., α>90° [23,51,76]. As pointed out in previous studies [23,76], this cross product rule may declare a topology violation based on a “false positive” when the minimum distance vector point passes through the vicinity of one of the bonded beads. It is possible in this case that the angle α exceeds 90° but no bond crossing occurs. These false cases are carefully excluded in the present analysis [23]. Figure 9 plots the relative number of topology violations per 100τ for semidilute solutions with different polymer fractions. As evident in Figure 9, the number of topology violations increases as φ increases. The concentrated solution at ϕ=0.7 generates topology violations 2.5 times as much as those in the dilute solutions. The inset of Figure 9 also demonstrates the number of topology violations occurring with a specific value of angle α. The results indicate that a majority of the bond crossings occur with a change in orientation by angles close to 180°. The distribution is consistent with the result of DPD simulations [75]. Importantly, the number of topology violations per 100τ in the MDPD simulations is on the order of 10^2^ (see Table 2), which is two orders of magnitude smaller than the values measured in the standard DPD simulations (~10^4^) [23,51]. This implies that the MDPD force law is more effective than the standard DPD force in preventing topology violations and capturing entanglements, which is attributed to the stronger repulsion between beads. Additional bond–bond repulsive interactions may be introduced to further reduce the number of topology violations [51,70,75,76]. 

## 4. Conclusions

This work focuses on modeling polymer solutions on the mesoscale using many-body dissipative particle dynamics. The polymer volume fraction of the solution is varied in a wide range from 0.008 to 0.7, corresponding to a gradual transition from dilute to semidilute regimes. A systematic parameterization of MDPD interactions is conducted for MDPD systems with liquid–vapor coexistence. By mapping onto the Flory–Huggins theory, a linear relation between the difference in MDPD attraction parameter Δa and the Flory–Huggins *χ* parameter is obtained, similar to previous DPD and MDPD studies. The surface tension of MDPD binary mixtures as a function of the *χ* parameter agrees well with the classical van der Waals theory, despite a higher critical point found from the fitting than the classical value.

The static and dynamic scaling relations are characterized for dilute polymer solutions, where the solvent quality is controlled by the polymer–solvent attraction parameter. Through the measurements of the radius of gyration and end-to-end distance, the scaling exponents indicate that polymer chains exhibit good solvent behavior in an athermal solution. The effective excluded volume interactions are induced by the density-dependent repulsion of the MDPD force law. The dynamic scalings of the diffusion coefficient and relaxation time of chains are consistent with the Zimm theory, which verifies the existence of hydrodynamic interactions in MDPD.

As the volume fraction increases and the solution enters the semidilute regime, the spatial overlapping of polymer coils results in the screening of excluded volume effect and the decrease of the radius of gyration. Meanwhile, the increasingly dominant inter-molecular interactions and chain entanglements significantly inhibit polymer dynamics. Due to the soft repulsion in MDPD, quantitative analysis is carried out to measure unphysical topology violations. The results confirm that entanglements are effective captured in the MDPD simulations.

Finally, it is noteworthy that the MDPD model of polymer solutions provides a powerful tool for efficiently simulating polymeric systems with liquid–vapor coexistence. This is particularly appealing for exploring the evaporation-driven dynamics of polymer chains and other inclusions on the mesoscale, which has significant implications for exerting better control on the processing of polymer thin films and polymer nanocomposites.

## Figures and Tables

**Table 1:** Structural properties of dilute polymer solutions in the theta solvent condition. The mean-square radius of gyration $\langle R_{g}^{2}\rangle$ and mean-square end-to-end distance $\langle R_{1N}^{2}\rangle$ were obtained by averaging over all chains in the system for each frame, and then averaged over 5000 frames with a separation of 1*τ* between consecutive frames.

N	$\langle R_{g}^{2}\rangle$	$\langle R_{1N}^{2}\rangle$	$\langle R_{1N}^{2}\rangle /\langle R_{g}^{2}\rangle$	$C_{n}$
5	0.43	2.22	5.23	1.14
8	0.74	4.12	5.57	1.21
10	0.95	5.41	5.68	1.23
20	2.06	12.06	5.85	1.30
30	3.20	18.89	5.90	1.34
40	4.36	25.87	5.94	1.36
50	5.39	31.71	5.89	1.32
80	8.74	52.64	6.03	1.37
100	11.21	66.91	5.97	1.38
140	15.82	92.87	5.8	1.37

**Table 2:** Average number of topology violations per 100*τ* for in semidilute solutions with different polymer volume fractions. The results are obtained in simulations having a total time of t=5000τ.

φ	0.02	0.05	0.1	0.2	0.3	0.4	0.5	0.6	0.7
$N_{\mathrm{TV}}$	169.7	168.7	191.3	203.6	237.4	274.3	310.9	376.0	432.8
